# The Application of Nanotechnology and Nanomaterials in Cancer Diagnosis and Treatment: A Review

**DOI:** 10.7759/cureus.29059

**Published:** 2022-09-11

**Authors:** Chinmay Kher, Sunil Kumar

**Affiliations:** 1 Department of Medicine, Jawaharlal Nehru Medical College, Wardha, IND

**Keywords:** nanotechnology, prognosis, treatment, diagnosis, cancer, nanomedicine

## Abstract

Cancer is one of the deadliest diseases worldwide in present times, with its incidence on a tremendous rise. It is caused by uncontrolled cell growth. Cancer therapies have advanced substantially, but there is a need for improvement in specificity and fear of systemic toxicity. Early detection is critical in improving patients' prognosis and quality of life, and recent advancements in technology, especially in dealing with biomaterials, have aided in that surge. Nanotechnology possesses the key to solving many of the downsides of traditional pharmaceutical formulations. Indeed, significant progress has been made in using customized nanomaterials for cancer diagnosis and treatment with high specificity, sensitivity, and efficacy. Nanotechnology is the integration of nanoscience into medicine by the use of nanoparticles. The advent of nanoscience in cancer diagnosis and treatment will help clinicians better assess and manage patients and improve the healthcare system and services. This review article gives an account of the clinical applications of nanoscience in the modern management of cancer, the different modalities of nanotechnology used, and the limitations and possible side effects of this new tool.

## Introduction and background

Nanotechnology, nicknamed "the manufacturing technology of the twenty-first century," allows us to manufacture a vast range of sophisticated molecular devices by manipulating matter on an atomic and molecular scale. These nanomaterials possess the ideal properties of strength, ductility, reactivity, conductance, and capacity at the atomic, molecular, and supramolecular levels to create useable devices and systems in a length range of 1-100 nm. The materials' physical, chemical, and mechanical characteristics differ fundamentally and profoundly at the nanoscale from those of individual atoms, molecules, or bulk material, which enables the most efficient atom alignment in a very tiny space. Nanotechnology allows us to build various intricate nanostructured materials by manipulating matter at the atomic and molecular scale in terms of strength, ductility, reactivity, conductance, and capacity [1,2].

"Nanomedicine" is the science and technology used to diagnose, treat, and prevent diseases. It is also used for pain management and to safeguard and improve people's health through nanosized molecules, biotechnology, genetic engineering, complex mechanical systems, and nanorobots [3]. Nanoscale devices are a thousand times more microscopic than human cells, being comparable to biomolecules like enzymes and their respective receptors in size. Because of this property, nanosized devices can interact with receptors on the cell walls, as well as within the cells. By obtaining entry into different parts of the body, they can help pick up the disease, as well as allow delivery of treatment to areas of the body that one can never imagine being accessible. Human physiology comprises multiple biological nano-machines. Biological processes that can lead to cancer also occur at the nanoscale. Nanotechnology offers scientists the opportunity to experiment on macromolecules in real time and at the earliest stage of disease, even when very few cells are affected. This helps in the early and accurate detection of cancer.

In a nutshell, the utility of the nanoscale materials for cancer is due to the qualities such as the ability to be functionalized and tailored to human biological systems (compatibility), the ability to offer therapy or act as a therapeutic agent, the ability to act as a diagnostic tool, the capability to penetrate various physiological barriers such as the blood-brain barrier, the capability to accumulate passively in the tumor, and the ability to aggressively target malignant cells.

Nanotechnology in cancer management has yielded various promising outcomes, including drug administration, gene therapy, monitoring and diagnostics, medication carriage, biomarker tracing, medicines, and histopathological imaging. Quantum dots (QDs) and gold nanoparticles are employed at the molecular level to diagnose cancer. Molecular diagnostic techniques based on these nanoparticles, such as biomarker discovery, can properly and quickly diagnose tumors. Nanotechnology therapeutics, such as nanoscale drug delivery, will ensure that malignant tissues are specifically targeted while reducing complications. Because of their biological nature, nanomaterials can cross cell walls with ease. Because of their active and passive targeting, nanomaterials have been used in cancer treatment for many years. This research looks at its applications in cancer diagnosis and therapy, emphasizing the technology's benefits and limitations [3-5]. The various uses of nanotechnology have been enumerated in the Table 1.

**Table 1:** Uses of nanotechnology

Diagnostic uses	Therapeutic uses
Dendrimers	Liposomes
Nanoshells	Polymeric micelles
Gold nanoparticles	Carbon nanotubes
	Quantum dots
	Dendrimers

## Review

Nanoparticles in cancer diagnosis

Early cancer detection is half the problem solved in the battle against cancer. X-ray, ultrasonography, CT, magnetic resonance imaging (MRI), and PET scan are the imaging techniques routinely used to diagnose cancer. Morphological changes in tissues or cells (histopathology or cytology) help in the final confirmation of cancer. These techniques detect cancer only after visible changes in tissues, by which time the cancer might have proliferated and caused metastasis. Another limitation of conventional imaging techniques is their failure to distinguish benign from malignant tumors. Also, cytology and histopathology cannot be employed as independent, sensitive tests to detect cancer at an early stage. With innovative molecular contrast media and materials, nanotechnology offers quicker and more accurate initial diagnosis, along with an ongoing assessment of cancer patient care [6].

Although nanoparticles are yet to be employed in actual cancer detection, they are currently being used in a range of medical screening tests. Gold nanoparticles are among the most commonly used in home test strips. A significant advantage of using nanoparticles for the detection of cancer is that they have a large surface area to volume ratio in comparison to their larger counterparts. This property ensures antibodies, aptamers, small molecules, fluorescent probes, polyethylene glycol (PEG), and other molecules cover the nanoparticle densely. This presents multiple binding ligands for cancer cells (multivalent effect of nanotools) and therefore increases the specificity and sensitivity of the bioassay [7,8]. Applications of nanotechnology in diagnosis are for the detection of extracellular biomarkers for cancer and for in vivo imaging. A good nanoprobe must have a long circulating time, specificity to the cancer tissue, and no toxicity to nearby tissue [9,10].

Detection of Biomarkers

Nanodevices have been studied to detect blood biomarkers and toxicity to healthy tissues nearby. These biomarkers include cancer-associated circulating tumor cells, associated proteins or cell surface proteins, carbohydrates or circulating tumor nucleic acids, and tumor-shed exosomes. Though it is well known that these biomarkers help to detect cancer at a preliminary stage, they also help to monitor the therapy and recurrence. They have limitations such as low concentrations in body fluids, variations in their levels and timings in different patients, and difficult prospective studies. These hurdles are overcome by nanotechnology, which offers high specificity and sensitivity. High sensitivity, specificity, and multiplexed measurements are all possible with nano-enabled sensors. To further illuminate a problem, next-generation gadgets combine capture with genetic analysis [11-15].

Imaging Using Nanotechnology

Nanotechnology uses nanoprobes that will accumulate selectively in tumor cells by passive or active targeting. The challenges faced are the interaction of nanoparticles with blood proteins, their clearance by the reticuloendothelial system, and targeting of tumors. Passive targeting suggests a preference for collecting the nanoparticles in the solid tumors due to extravasation from the blood vessels. This is made possible by the defective angiogenesis of the tumor wherein the new blood vessels do not have tight junctions in their endothelial cells and allow the leaking out of nanoparticles up to 150 nm in size, leading to a preferential accumulation of nanoparticles in the tumor tissue. This phenomenon is called enhanced permeability and retention (EPR). Active targeting involves the recognition of nanoparticles by the tumor cell surface receptors. This will enhance the sensitivity of in vivo tumor detection. For early detection of cancer, active targeting will give better results than passive targeting [16-18].

Nanoparticles in cancer therapy

This can be classified as delivery of chemotherapy, immunotherapy, radiotherapy, and gene therapy, and delivery of chemotherapy is aimed at improving the pharmacokinetics and reducing drug toxicity by selective targeting and delivery to cancer tissues. This is primarily based on passive targeting, which employs the EPR effect described earlier [16]. Nanocarriers increase the half-life of the drugs. Immunotherapy is a promising new front in cancer treatment based on understanding the tumor-host interaction. Nanotechnology is being investigated to deliver immunostimulatory or immunomodulatory molecules. It can be used as an adjuvant to other therapies [19-21].

Role of Nanotechnology in Radiotherapy

This technology involves targeted delivery of radioisotopes, targeted delivery of radiosensitizer, reduced side effects of radiotherapy by decreasing distribution to healthy tissues, and combining radiotherapy with chemotherapy to achieve synergism but avoid side effects, and administering image-guided radiotherapy improves precision and accuracy while reducing exposure to surrounding normal tissues [22,23].

Gene Therapy Using Nanotechnology

There is a tremendous interest in the research in gene therapy for cancer, but the results are still falling short of clinical application. Despite a wide array of therapies aimed at gene modulation, such as gene silencing, anti-sense therapy, RNA interference, and gene and genome editing, finding a way to deliver these effects is challenging. Nanoparticles are used as carriers for gene therapy, with advantages such as easy construction and functionalizing and low immunogenicity and toxicity. Gene-targeted delivery using nanoparticles has great future potential. Gene therapy is still in its infancy but is very promising [24].

Nanodelivery Systems

Quantum dots: Semiconductor nanocrystal quantum dots (QDs) have outstanding physical properties. Probes based on quantum dots have achieved promising cellular and in vivo molecular imaging developments. Increasing research is proving that technology based on quantum dots may become an encouraging approach in cancer research [4]. Biocompatible QDs were launched for mapping cancer cells in vitro in 1998. Scientists used these to create QD-based probes for cancer imaging that were conjugated with cancer-specific ligands, antibodies, or peptides. QD-immunohistochemistry (IHC) has more sensitivity and specificity than traditional immunohistochemistry (IHC) and can accomplish measurements of even low levels, offering considerably higher information for individualized management. Imaging utilizing quantum dots has emerged as a promising technology for early cancer detection [25,26].

Nanoshells and gold nanoparticles/gold nanoshells (AuNSs) are an excellent example of how combining nanoscience and biomedicine can solve a biological problem. They have an adjustable surface plasmon resonance, which can be set to the near-infrared to achieve optimal penetration of tissues. During laser irradiation, AuNSs' highly effective light-to-heat transition induces thermal destruction of the tumor without harming healthy tissues. AuNSs can even be used as a carrier for a wide range of diagnostic and therapeutic substances [27].

Dendrimers: These are novel nanoarchitectures with distinguishing characteristics such as a spherical three-dimensional shape, a monodispersed uni-micellar nature, and a nanometric size range. The biocompatibility of dendrimers has been employed to deliver powerful medications such as doxorubicin. This nanostructure targets malignant cells by attaching ligands to their surfaces. Dendrimers have been intensively investigated for targeting and delivering cancer therapeutics and magnetic resonance imaging contrast agents. The gold coating on its surface significantly reduced their toxicity without significantly affecting their size. It also served as an anchor for attaching high-affinity targeting molecules to tumor cells [28].

**Figure 1:**
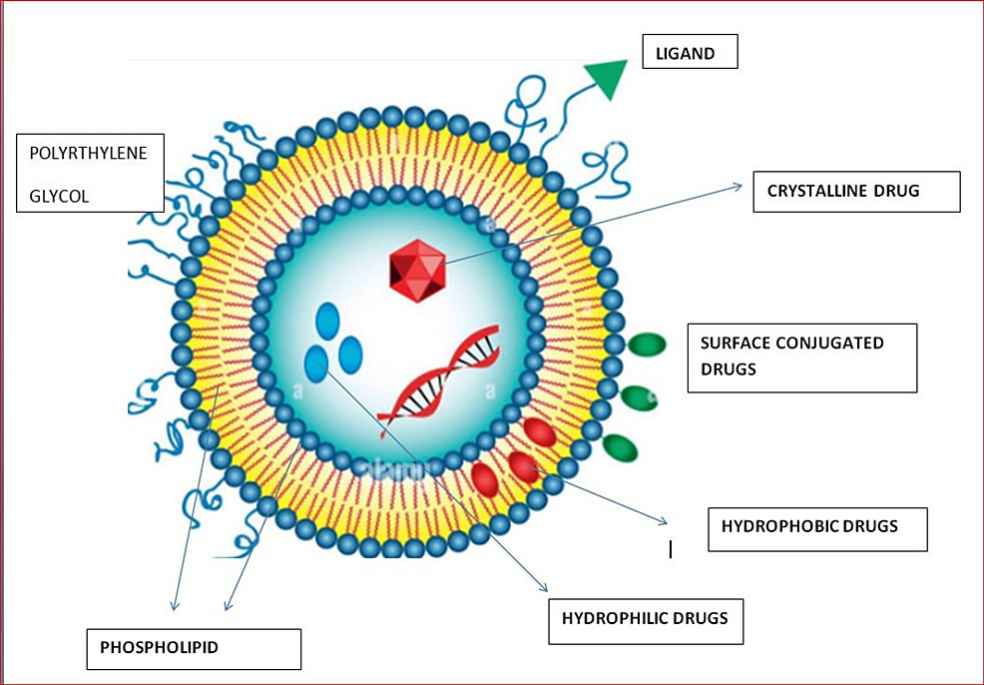
Liposomes Author's own creation

Liposomal nanoparticles (Figure 1): These have a role in delivery to a specific target spot, reducing biodistribution toxicity because of the surface-modifiable lipid composition, and have a structure similar to cell membranes. Liposome-based theranostics (particles constructed for the simultaneous delivery of therapeutic and diagnostic moieties) have the advantage of targeting specific cancer cells. Liposomes are more stable in the bloodstream and increase the solubility of the drug. They also act as sustained release preparations and protect the drug from degradation and pH changes, thereby increasing the drug's circulating half-life. Liposomes help to overcome multidrug resistance. Drugs such as doxorubicin, daunorubicin, mitoxantrone, paclitaxel, cytarabine, and irinotecan are used with liposome delivery [29-31].

Polymeric micelles: Micelles are usually spherical particles with a diameter of 10-100 nm, which are self-structured and have a hydrophilic covering shell and a hydrophobic core, suspended in an aqueous medium. Hydrophobic medicines can be contained in the micelle's core. A variety of molecules having the ability to bind to receptors, such as aptamers, peptides, antibodies, polysaccharides, and folic acid, are used to cover the surface of the micelle in active tumor cell targeting. Enzymes, ultrasound, temperature changes, pH gradients, and oxidation are used as stimuli in micelle drug delivery systems. Various physical and chemical triggers are used as stimuli in micelle drug delivery systems. pH-sensitive polymer micelle is released by lowering pH. A co-delivery system transports genetics, as well as anticancer medicines. Although paclitaxel is a powerful microtubule growth inhibitor, it has poor solubility, which causes fast drug aggregation and capillary embolisms. Such medicines' solubility can be raised to 0.0015-2 mg/ml by encapsulating them in micelles. Polymeric micelles are now being tested for use in nanotherapy [32].

Carbon nanotubes (CNTs): Carbon from burned graphite is used to create hollow cylinders known as carbon nanotubes (CNTs). They possess distinct physical and chemical characteristics that make them interesting candidates as carriers of biomolecules and drug delivery transporters. They have a special role in transporting anticancer drugs with a small molecular size. Wu et al. formed a medicine carrier system using multi-walled CNTs (MWCNTs) and the 10-hydroxycamptothecin (HCPT) anticancer compound. As a spacer between MWCNTs and HCPT, they employed hydrophilic diamine trimethylene glycol. In vitro and in vivo, their HCPT-MWCNT conjugates showed significantly increased anticancer efficacy when compared to traditional HCPT formulations. These conjugates were able to circulate in the blood longer and were collected precisely at the tumor site [33,34].

Limitations

Manufacturing costs, extensibility, safety, and the intricacy of nanosystems must all be assessed and balanced against possible benefits. The physicochemical properties of nanoparticles in biological systems determine their biocompatibility and toxicity. As a result, stringent manufacturing and delineation of nanomaterials for delivery of anticancer drugs are essential to reduce nanocarrier toxicity to surrounding cells. Another barrier to medication delivery is ensuring public health safety, as issues with nanoparticles do not have an immediate impact. The use of nanocarriers in cancer treatment may result in unforeseen consequences. Hypothetical possibilities of environmental pollution causing cardiopulmonary morbidity and mortality, production of reactive oxygen species causing inflammation and toxicity, and neuronal or dermal translocations are a few possibilities that worry scientists. Nanotoxicology, a branch of nanomedicine, has arisen as a critical topic of study, paving the way for evaluating nanoparticle toxicity [35-37].

## Conclusions

Nanotechnology has been one of the recent advancements of science that not only has revolutionized the engineering field but also is now making its impact in the medical and paramedical field. Scientists have been successful in knowing the properties and characteristics of these nanomaterials and optimizing them for use in the healthcare industry. Although some nanoparticles have failed to convert to the clinic, other new and intriguing nanoparticles are now in research and show great potential, indicating that new treatment options may be available soon. Nanomaterials are highly versatile, with several benefits that can enhance cancer therapies and diagnostics.

These are particularly useful as drug delivery systems due to their tiny size and unique binding properties. Drugs such as doxorubicin, daunorubicin, mitoxantrone, paclitaxel, cytarabine, irinotecan, and amphotericin B are already being conjugated with liposomes for their delivery in current clinical practices. Doxorubicin, cytarabine, vincristine, daunorubicin, mitoxantrone, and paclitaxel, in particular, are key components of cancer chemotherapy. Even in the diagnosis of cancer for imaging and detection of tumor markers, particles such as nanoshells, dendrimers, and gold nanoparticles are currently in use.

Limitations of this novel technology include manufacturing expenses, extensibility, intricacy, health safety, and potential toxicity. These are being overcome adequately by extensive research and clinical trials, and nanomedicine is becoming one of the largest industries in the world. A useful collection of research tools and clinically practical gadgets will be made available in the near future thanks to advancements in nanomedicine. Pharmaceutical companies will use in vivo imaging, novel therapeutics, and enhanced drug delivery technologies in their new commercial applications. In the future, neuro-electronic interfaces and cell healing technology may change medicine and the medical industry when used to treat brain tumors.
